# A Rare Combination of Complex Elbow Dislocation and Distal Radial Fracture in Adults

**DOI:** 10.7759/cureus.868

**Published:** 2016-11-08

**Authors:** Raju Vaishya, Midhun Krishnan, Vipul Vijay, Amit Kumar Agarwal

**Affiliations:** 1 Orthopaedics, Indraprastha Apollo Hospitals; 2 Orthopaedics, Medical College and Hospital, Karakonam, Trivandrum, Kerala

**Keywords:** elbow, dislocation, complex, radius, fractures, mechanism

## Abstract

Although it is common for separate elbow joint dislocation and fracture of forearm bones to occur, it is a rare sighting for both elbow dislocation and ipsilateral fracture of the distal radius. We report a case of an anterior dislocation of the elbow with ipsilateral fracture of the distal radius. The case was treated operatively. We describe two possible mechanisms of injury for these rare injury types. The case underlines the importance of assessing the wrist in the case of an elbow fracture and vice versa.

## Introduction

The elbow is the second most common joint affected by dislocation. These dislocations can be divided into simple and complex, depending on their association with a fracture. A simple dislocation is a pure capsuloligamentous trauma without any associated fracture. When the dislocation is associated with a fracture, it is classified as a complex dislocation. The association of a fracture with an elbow dislocation is about 20% [1]. Moreover, the most common variety of elbow dislocation is posterior, and anterior elbow dislocations are extremely rare. Complex elbow dislocations usually involve the proximal part of the ulna and radius [1]. The involvement of the distal radius with an elbow dislocation is rare [2]. Only a few case reports of posterior elbow dislocation along with ipsilateral radius fracture have been reported in the literature [3-4]. We describe a case of elbow dislocation associated with a distal end radius fracture and discuss possible mechanisms of action.

Informed consent was obtained by the patient for treatment.

## Case presentation

A 55-year-old female presented to the emergency department with a history of fall on her outstretched hand after falling from a height of seven feet. She had complaints of pain over her left elbow joint and wrist joint. On physical examination, her left elbow was grossly deformed, and there was swelling over her left wrist. Tenderness was present over both her left elbow and wrist joints. The range of motion of her left wrist and left elbow joint were painfully restricted. No distal neurovascular deficit was noted.

**Figure 1:**
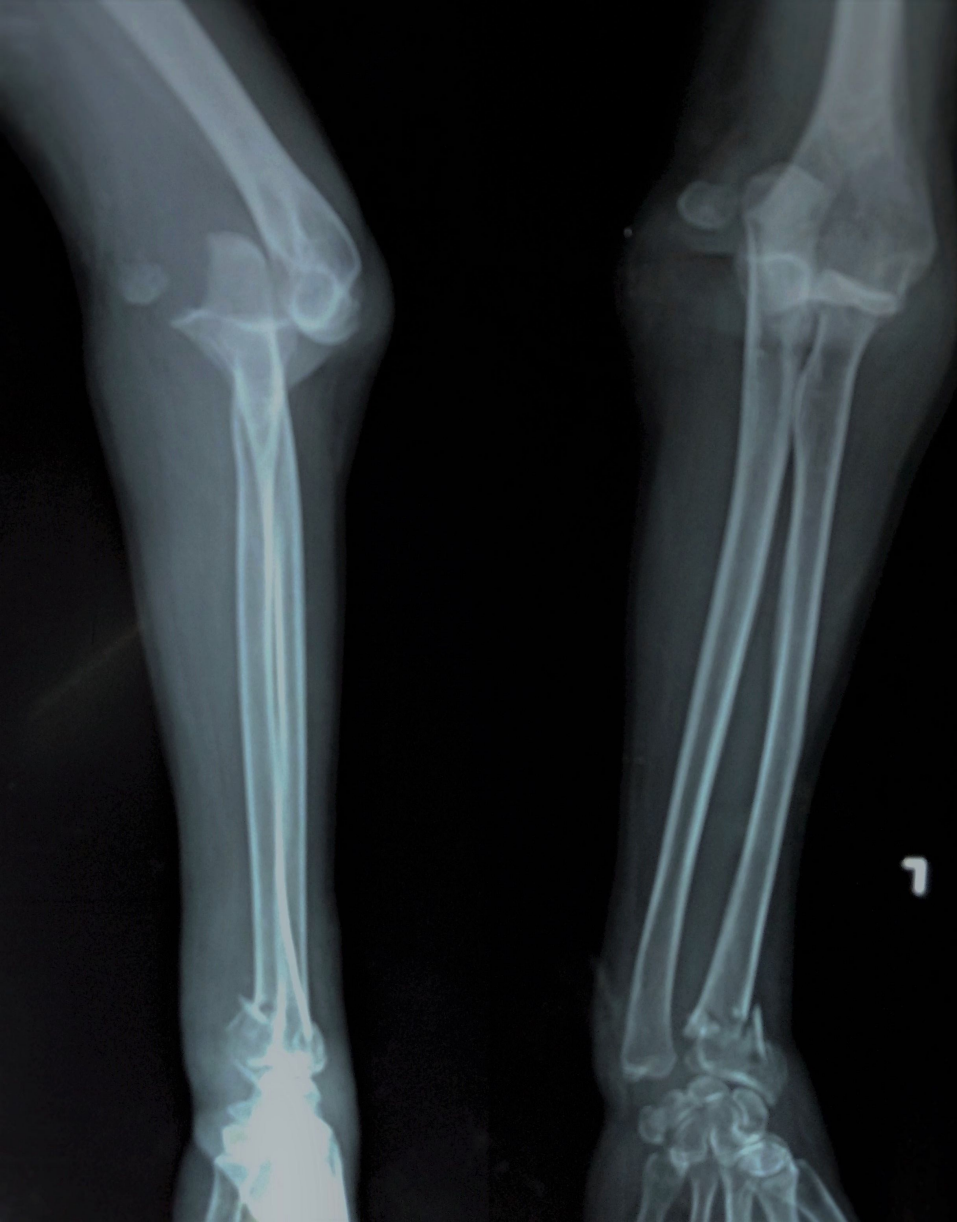
Anterior elbow dislocation along with the lateral epicondyle fracture.

Her plain x-rays revealed an anterior elbow dislocation and distal radius fracture with intra-articular extension. There was an associated displaced fracture of the lateral epicondyle (Figure 1).

The elbow joint was reduced under general anesthesia. A distal traction was given at the forearm. Since there was a fracture at the distal end of the radius as well, the traction could not be applied at the wrist. The traction was thus applied to the proximal forearm, and a posteriorly directed force was applied while the elbow was hyperflexed. Care was taken not to hyperextend the elbow joint as it could have put the anterior neurovascular bundle at a stretch. The elbow joint was unstable post reduction and, hence, was fixed with two ulnohumeral pins.

**Figure 2:**
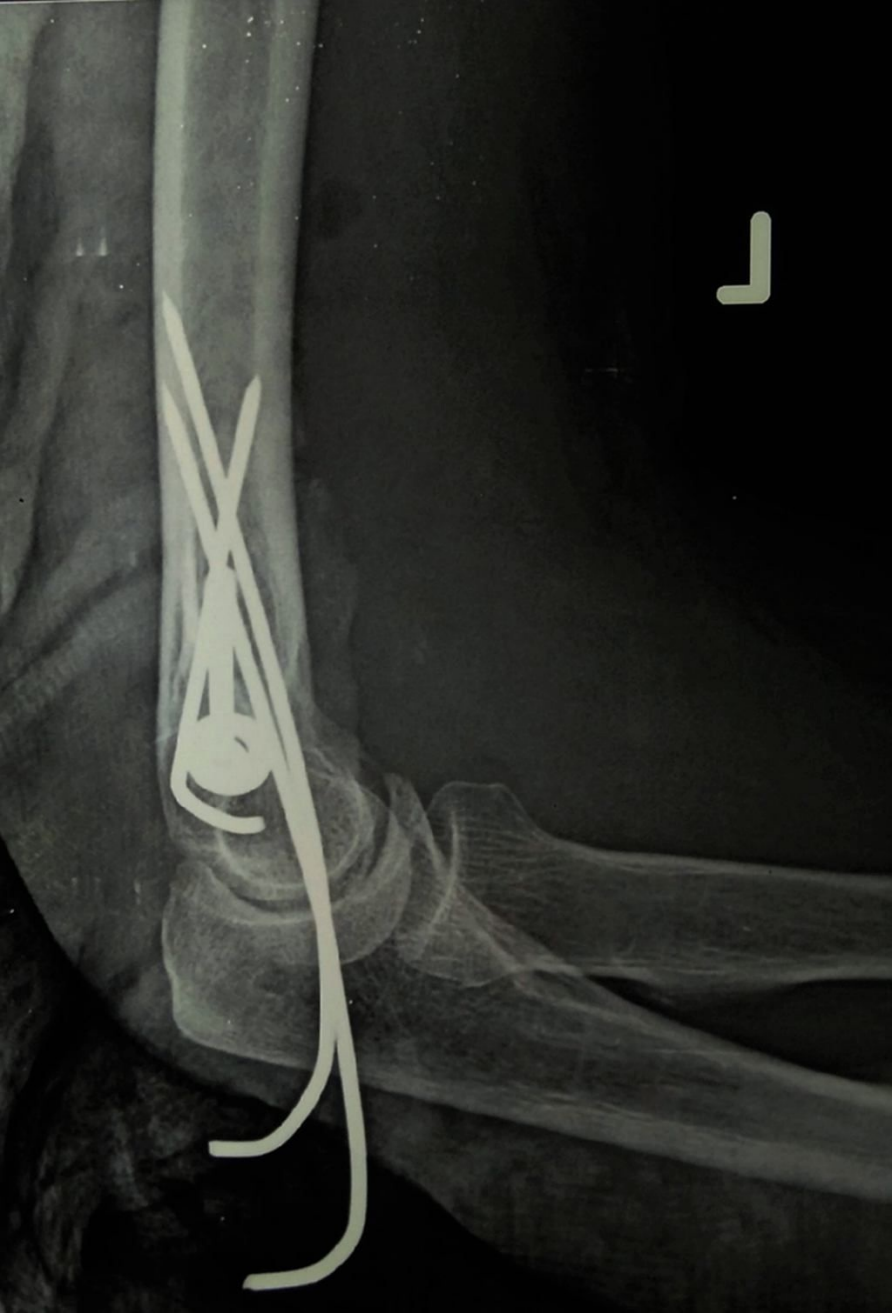
Fixation of the elbow done with two ulnohumeral pins and a partially threaded cannulated cancellous screw with a washer.

The displaced lateral epicondyle fracture was accessed through the lateral approach to the elbow and fixed with a partially threaded cannulated cancellous screw with a washer (Figure 2).

**Figure 3:**
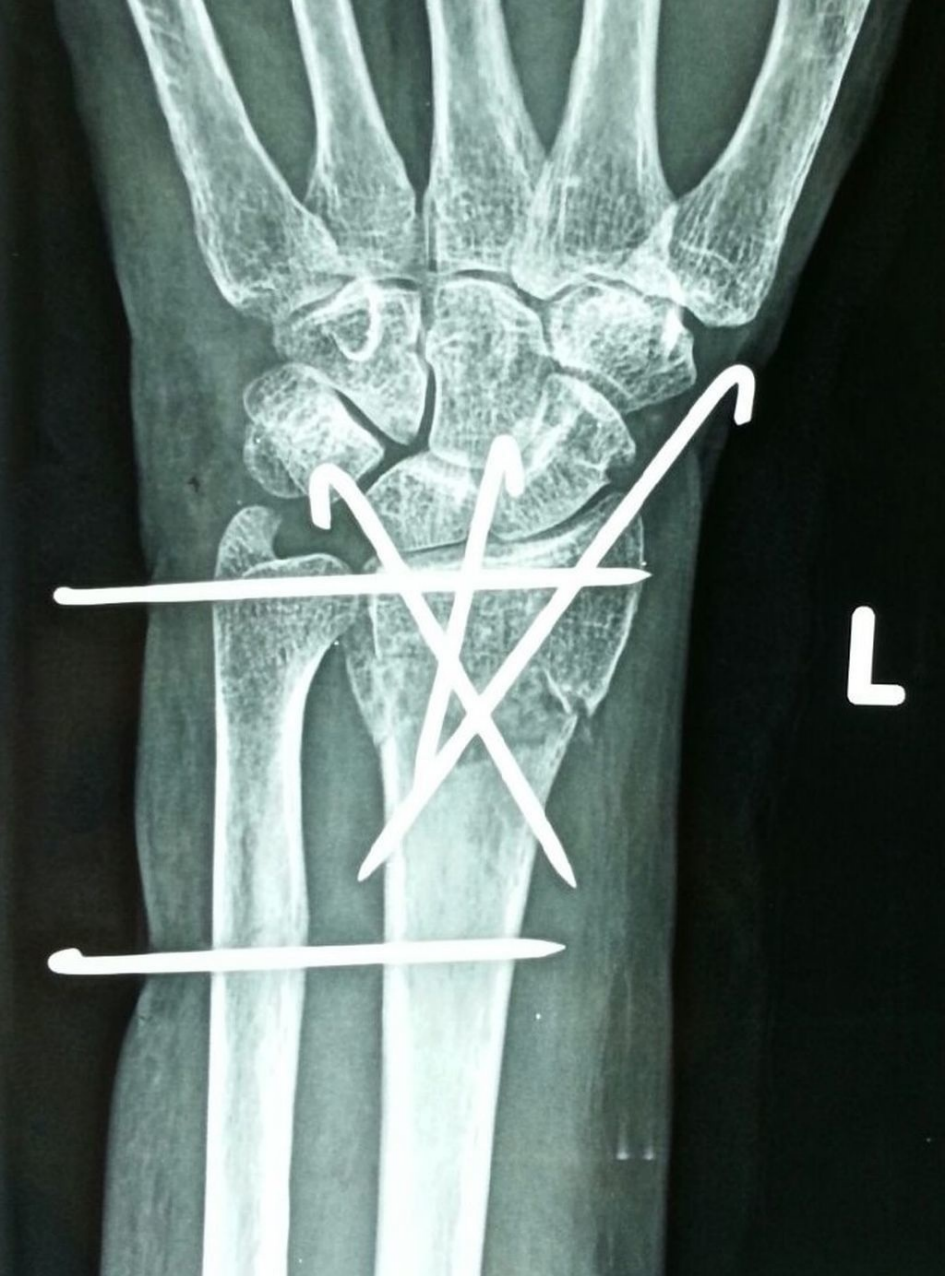
Left distal radius pinning - anteroposterior view.

The distal radius fracture was then reduced and pinned using five percutaneous K-wires. Two 1.5 mm K-wires were inserted from distal to proximal, and another pin was passed through the radial styloid. Two K-wires were added from the ulnar side to the radial side for prevention of supination and pronation (Figure 3).

**Figure 4:**
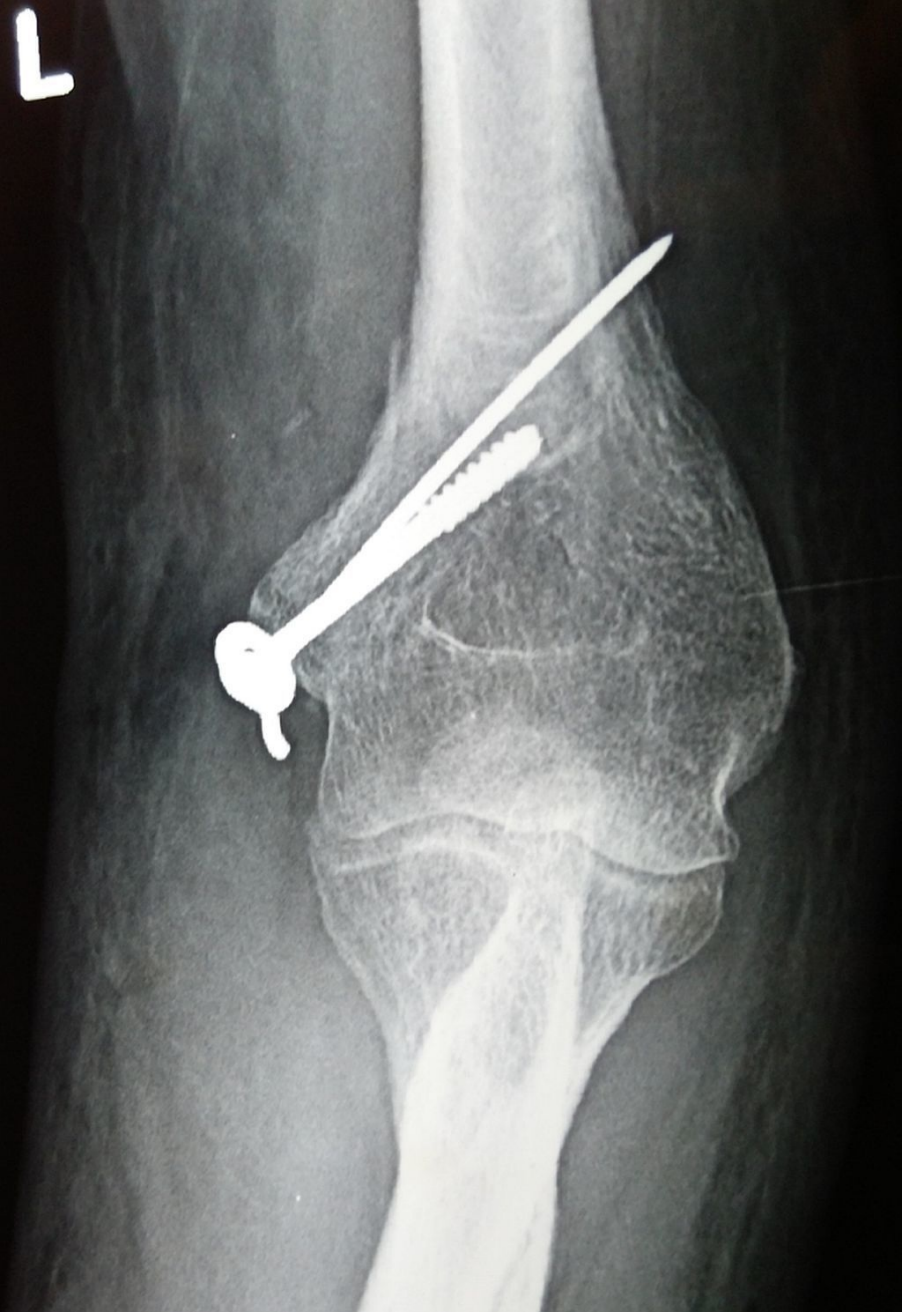
Anteroposterior radiograph of the elbow showing a reduced elbow joint with the screw and K-wire in situ.

An above-elbow cast was applied for two weeks. Following this, the ulnohumeral pins along with the slab were removed, and a wrist brace was given. The patient was reviewed weekly for her pin site dressings, and elbow mobilization was encouraged. The wrist splint was then removed after four more weeks, and active elbow and wrist movements were started six weeks after trauma. At the three-month follow-up, the lateral epicondyle fracture had united, and the patient had regained flexion range of motion of zero degrees to 130 degrees at the elbow (Figure 4).

**Figure 5:**
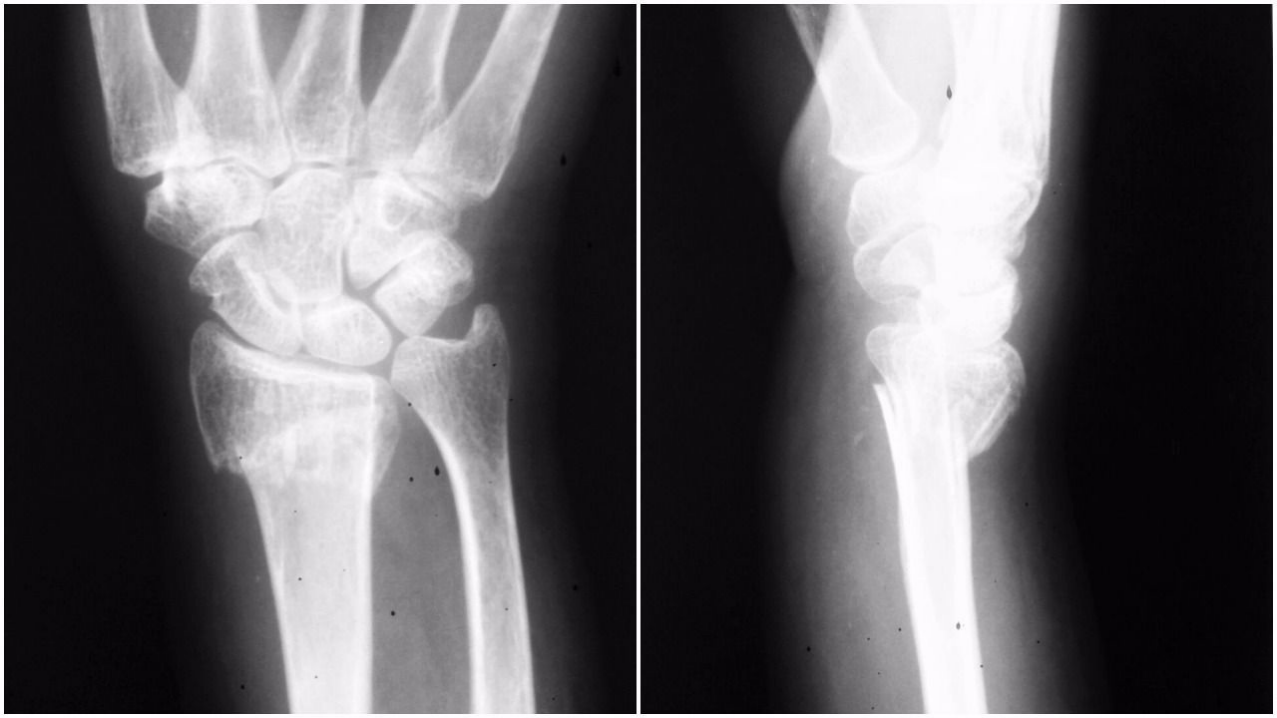
Three-month postoperative x-ray of left distal radius - anteroposterior and lateral view.

The wrist had dorsiflexion of 70 degrees, palmar flexion of 40 degrees, and supination restriction of 10 degrees as compared to the normal side. At the final follow-up of one year, the patient was asymptomatic with both fractures united and a congruent elbow joint. The distal end radius fracture had malunited but was asymptomatic (Figure 5.)

## Discussion

The elbow joint is a stable joint of the hinged variety. Radial head, coronoid process, and olecranon fractures are most commonly associated with elbow dislocations [2]. Fracture of the radial head and coronoid process along with posterior dislocation is called the "terrible triad" [1]. Radial head fracture along with an ulnar diaphyseal fracture has been described as Monteggia fracture dislocation [5]. Elbow dislocations with ipsilateral radial and ulnar diaphyseal fractures have also been reported in the literature [6].There have also been reported cases of Galeazzi-variant type fracture dislocation injury in adults [7].

The association of elbow dislocation with distal radius fracture is rare. The cases that are already reported in this pattern involve those in the pediatric age group, those with a compound fracture, and a few cases of a combination of posterior elbow dislocation with ipsilateral distal radius fractures [3-4, 8-10]. The association of the distal end radius fracture with an anterior elbow dislocation has not been reported in the literature. A simple posterior elbow dislocation most commonly describes a fall on the outstretched hand as the mechanism of injury, while that of the simple anterior elbow dislocation is usually a direct blow to the elbow. Also, a simple posterior elbow dislocation occurs when it is slightly abducted and flexed.

**Figure 6:**
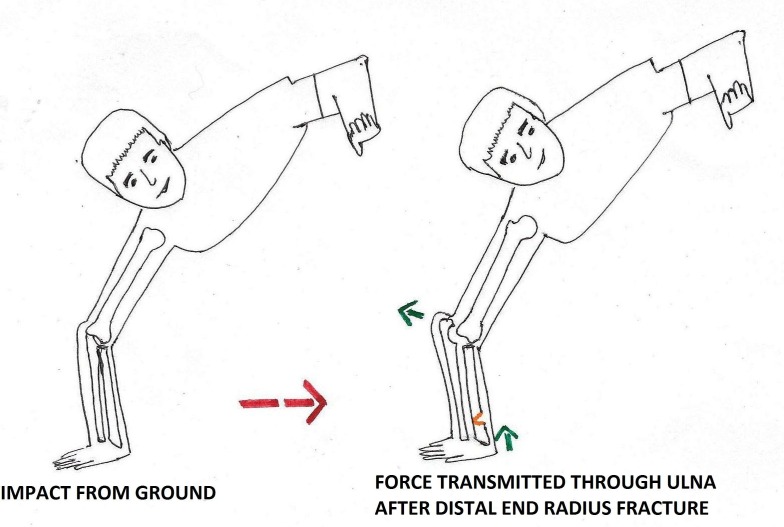
Mechanism of anterior elbow dislocation with an ipsilateral distal radius fracture.

We would like to propose two mechanisms of injury for dislocation of the elbow with an ipsilateral distal radius fracture. The first mechanism would be a single-impact theory of a fall from height resulting in compressive forces that, when directed on the outstretched hand, fracture the distal end of the radius first. Since the energy is enormous, it travels to the hyperextended and valgus elbow, resulting in dislocation of the elbow posteriorly. Since the force fractures the radial column, the remaining force is only transmitted along the ulna, thus pushing the ulnar groove out of the trochlea and, hence, causing posterior dislocation. The second mechanism for the above injury pattern would be a double-impact theory as seen in road traffic injury patients. In this arrangement, the subject undergoes a fall on the outstretched hand resulting in the distal radius fracture, followed by a direct injury to the elbow resulting in the posterior elbow dislocation (Figure 6).

**Figure 7:**
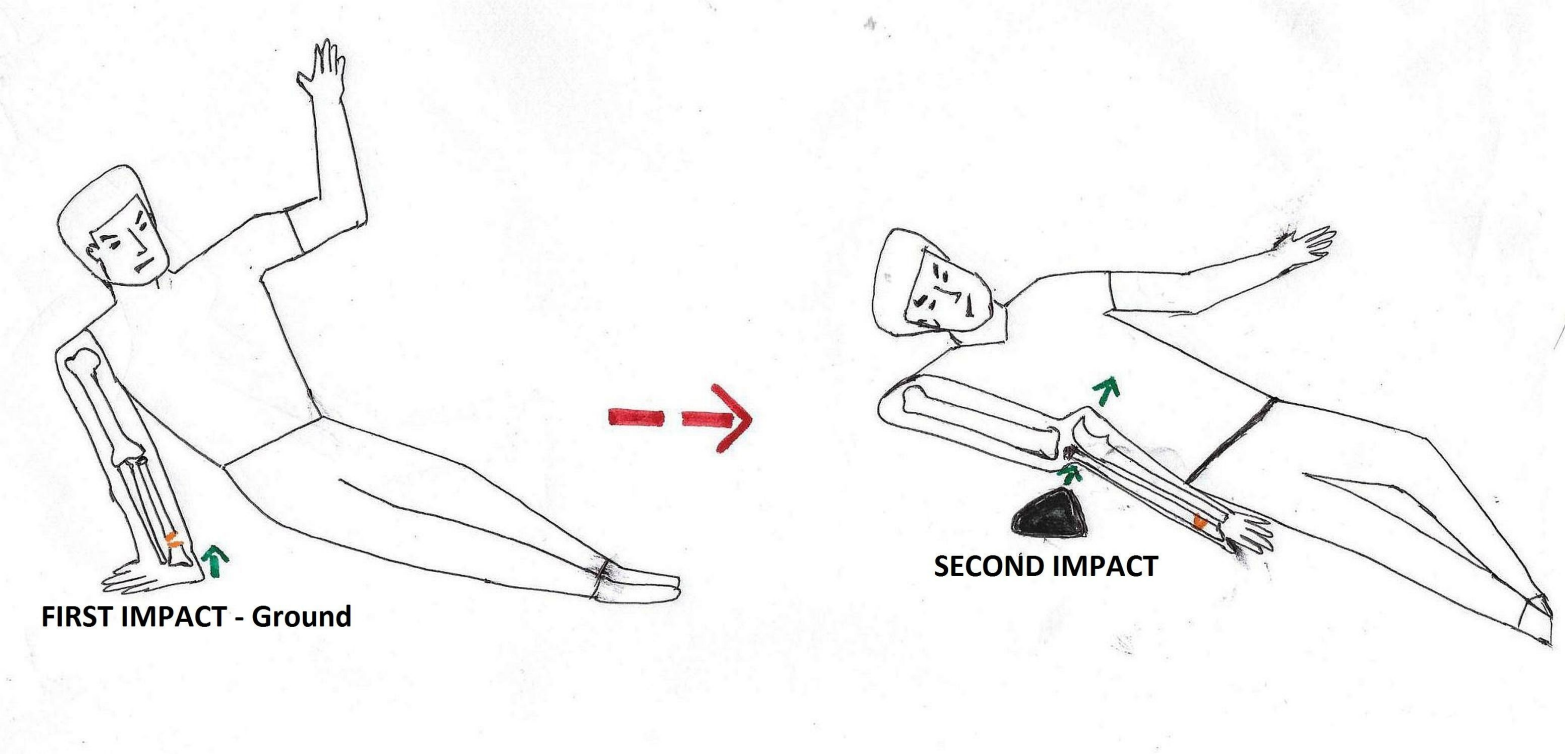
Mechanism of single-impact theory of posterior elbow dislocation with an ipsilateral distal radius fracture.

We believe that the most probable mechanism of injury of the anterior dislocation with an ipsilateral distal radius fracture would be a double-impact theory of a fall on the outstretched hand from height causing the distal radius to fracture while landing on the ground, followed by a direct posterior blow to a flexed elbow (Figure 7).

Elbow dislocation can be reduced under sedation, but in our case, the reduction was found to be unstable and, hence, post reduction ulnohumeral pinning had to be done. Following this, with the elbow in 90 degrees of flexion, the distal radius was reduced, and pinning of the distal radius fracture was done. In this case, even though the fracture was also amenable to plate osteosynthesis, closed reduction and pinning were done due to financial constraints. The distal end radius fracture malunited, but the malunion was asymptomatic until the last follow-up.

The purpose of this study is to report that there is a chance of double site, closed type of fracture in the forearm in cases of a fall from height. A thorough investigation of these injuries should be done as these can be easily missed. The radiographs of an elbow injury case should always include that of the wrist and vice versa.

## Conclusions

Elbow dislocation with ipsilateral distal radius fracture is rare. A considerable force must be applied to cause this pattern of injury, such as that of a fall from height. Our case reiterates the point that in every elbow dislocation case, the wrist joints need to be evaluated both clinically and radiologically. Also, these injuries can be classified into two types: (1) an anterior elbow dislocation with an ipsilateral distal radius fracture and (2) a posterior elbow dislocation with an ipsilateral wrist dislocation. The mechanism of reduction of the elbow joint would vary with the direction of dislocation. Such injuries should be expected when the injury pattern matches the described mechanism.
